# Retraction: NAD(P)H: Quinone oxidoreductase 1 deficiency conjoint with marginal vitamin C deficiency causes cigarette smoke induced myelodysplastic syndromes

**DOI:** 10.1371/journal.pone.0340144

**Published:** 2026-01-06

**Authors:** The PLOS One Editors

After this article [1] was published, concerns were raised regarding results presented in Figs 3, 4, S1, and S2, and in Table S2.

Specifically:

- The following panels appear similar despite representing different experimental results:
  - Fig 3A 7 Days 15mg vit C TUNEL and 21 Days 15mg vit C TUNEL
  - Fig 3G α-tubulin, Fig 4A α-tubulin, and Fig S1A α-tubulin
- In Fig 4A lanes 7-8 central panel, there appear to be areas of the background that are discontinuous with the surrounding areas
- In Fig S2A, panels III and IV appear to partially overlap
- The Table S2A 15 mg vit C Air and the Table S2B 0.5 mg vit C DC + CS 0 Days results show the same mean Myeloid: Nonmyeloid ratio and standard deviation
- In the following western blot panels, the marker “M” lanes appear similar: Fig 4A and Fig 4B in [1], and Fig 2A, Fig 2B, Fig 6B, and Fig 6C in [2, retracted in 3]

Regarding Fig 3A, the first author stated the 21 Days 15 mg vit C TUNEL panel is incorrect.

The first author stated they agree with the concerns for the α-tubulin panels in Figs 3G, 4A, and S1A. They stated that an error occurred in the figure preparation and that Fig 4A α-tubulin is incorrect and provided a replacement panel.

Regarding the marker lanes in Fig 4 in [1] and Figs 2 and 6 in [2], the first author stated these lanes are similar because the experiments were run under similar percentage SDS-PAGE and the same voltage, and the protein marker is shown for reference only. They provided data underlying Figs 4A and 4B, however editorial review raised additional concerns about the integrity of the underlying data provided.

The first author provided incomplete underlying data for Figs 3, 4, S1, S2 and Table S2. These data did not resolve the above concerns, and PLOS identified additional concerns with the provided data underlying Table S2.

In light of the above concerns that question the reliability of the reported results, the *PLOS One* Editors retract this article.

AD did not agree with the retraction. ND, AG, and TD either did not respond directly or could not be reached. IBC is deceased.
